# An automated approach for real-time informative frames classification in laryngeal endoscopy using deep learning

**DOI:** 10.1007/s00405-024-08676-z

**Published:** 2024-05-02

**Authors:** Chiara Baldini, Muhammad Adeel Azam, Claudio Sampieri, Alessandro Ioppi, Laura Ruiz-Sevilla, Isabel Vilaseca, Berta Alegre, Alessandro Tirrito, Alessia Pennacchi, Giorgio Peretti, Sara Moccia, Leonardo S. Mattos

**Affiliations:** 1 Department of Advanced Robotics, Istituto Italiano di Tecnologia, Genoa, Italy; 2 Departement of Informatics, Bioengineering, Robotics and Systems Engineering, University of Genoa, Genoa, Italy; 3 Department of Experimental Medicine (DIMES), University of Genoa, Genoa, Italy; 4 Department of Otolaryngology, Hospital Clínic, C. de Villarroel, 170, 08029 Barcelona, Spain; 5 Unit of Head and Neck Tumors, Hospital Clínic, Barcelona, Spain; 6 Unit of Otolaryngology, Trento, Italy; 7 Otorhinolaryngology Head-Neck Surgery Department, Hospital Universitari Joan XXIII de Tarragona, Tarragona, Spain; 8 Translational Genomics and Target Therapies in Solid Tumors Group, Institut d́Investigacions Biomèdiques August Pi i Sunyer, IDIBAPS, Barcelona, Spain; 9 Faculty of Medicine, University of Barcelona, Barcelona, Spain; 10 Unit of Otorhinolaryngology-Head and Neck Surgery, IRCCS Ospedale Policlinico San Martino, Genoa, Italy; 11 Department of Surgical Sciences and Integrated Diagnostics (DISC), University of Genoa, Genoa, Italy; 12 The BioRobotics Institute and Department of Excellence in Robotics and AI, Scuola Superiore Sant’Anna, Pisa, Italy

**Keywords:** Laryngeal cancer, Laryngoscopy, Artificial intelligence, Deep learning, Larynx

## Abstract

**Purpose:**

Informative image selection in laryngoscopy has the potential for improving automatic data extraction alone, for selective data storage and a faster review process, or in combination with other artificial intelligence (AI) detection or diagnosis models. This paper aims to demonstrate the feasibility of AI in providing automatic informative laryngoscopy frame selection also capable of working in real-time providing visual feedback to guide the otolaryngologist during the examination.

**Methods:**

Several deep learning models were trained and tested on an internal dataset (n = 5147 images) and then tested on an external test set (n = 646 images) composed of both white light and narrow band images. Four videos were used to assess the real-time performance of the best-performing model.

**Results:**

ResNet-50, pre-trained with the pretext strategy, reached a precision = 95% vs. 97%, recall = 97% vs, 89%, and the F1-score = 96% vs. 93% on the internal and external test set respectively (p = 0.062). The four testing videos are provided in the supplemental materials.

**Conclusion:**

The deep learning model demonstrated excellent performance in identifying diagnostically relevant frames within laryngoscopic videos. With its solid accuracy and real-time capabilities, the system is promising for its development in a clinical setting, either autonomously for objective quality control or in conjunction with other algorithms within a comprehensive AI toolset aimed at enhancing tumor detection and diagnosis.

**Supplementary Information:**

The online version contains supplementary material available at 10.1007/s00405-024-08676-z.

## Introduction

High-definition videoendoscopy and laryngoscopy are nowadays the cornerstones of laryngeal examination. The use of Narrow Band Imaging (NBI) optical modality, as opposed to standard White Light (WL) endoscopy, has been demonstrated to offer benefits in enhancing the visualization of vessel abnormalities and characterization of diseases [1, 2]. Traditionally, the quality of the endoscopic evaluation has relied on the subjective expertise of medical practitioners, resulting in inherent inter- and intra-observer variability. Due to time constraints and patient compliance, often a clear view of the larynx is not obtained, with possible misdetection of abnormal laryngeal conditions. Conversely, when a lesion is found, if the quality of the view is not optimal, the risk of misdiagnosis increases with a consequent potential undervaluation of malignant disorders, ultimately compromising patient care and management.

The advent of Artificial Intelligence (AI) has transformed numerous domains within the medical field, ushering in an era of unprecedented precision and efficiency [3]. Specifically, its application in medical imaging can be exploited for analyzing a vast amount of information, provided by videos and images, with the potential to enhance diagnostic accuracy and streamline clinical workflows. The integration of AI technologies with the imaging field is called computer vision, and especially in the upper aerodigestive tract endoscopy, such technology has demonstrated unprecedented power for automating and standardizing the analysis of laryngoscopic images [4]. Particularly, several methods have been proposed during the last years for automated key frame extraction in endoscopic videos [5–8]. The most popular approach in this field is deep learning (DL) which allows computer systems to automatically learn how to recognize images by leveraging vast datasets of annotated laryngoscopy images.

These solutions can have an important impact in the otolaryngological practice. First, the automatic extraction of frames from endoscopic videos can assist the development of computer-aided detection [9], segmentation [10, 11], and Diagnosis systems [12], as their outcomes rely on substantial amounts of well-annotated data. From the clinical point of view, standardizing the analysis of laryngoscopic data can enhance the accuracy of endoscopic diagnosis and facilitate the clinical decision-making process by automatically selecting and saving the keyframes with the optimal level of information, ultimately saving time when reviewing the examination. Finally, during the outpatient examination of the larynx, the implementation of a method for recognizing informative frames might become a way to give otorhinolaryngologists real-time feedback about how to adjust the navigation of endoscopic tools within the target or to direct their attention toward data that clearly depict the laryngeal structures.

In this paper, we explore the development and validation of a DL model designed to face the classification of informative frames in laryngoscopy. The contributions of this research are two-fold: first, we aim to demonstrate the feasibility and efficacy of AI in providing automatic informative laryngoscopy frame selection both in WL and NBI; second, by pursuing a real-time computing algorithm, we strive to establish the groundwork for its seamless integration into clinical practice with the aim of providing visual feedback to guide the otolaryngologist during the examination.

## Materials and methods

### Study population and image datasets

The “NBI-InfFrames” from Moccia et al. [8], which to our knowledge is the only publicly available dataset to date, was initially used for this study. It comprises 720 frames from NBI videos of n = 18 patients affected by laryngeal Squamous Cell Carcinoma: 180 informative frames with proven good-quality information, 180 dark frames identified as underexposed, 180 blurred frames, and 180 frames that exhibit either saliva or specular reflections. The images were captured with a resolution of 1920 × 1072 pixels, using an Olympus Visera Elite S190 video processor and an ENF- VH video rhino-laryngo video scope (Olympus Medical System Corporation, Tokyo, Japan). However, the dataset presents some weaknesses: it consists of only NBI frames and it is based only on a few patients, which might reduce the generalization capability of a model trained only on this dataset. Finally, prospectively considering screening campaigns, the informative frame selection should be exploited not only for patients with laryngeal carcinoma but also for other diseases and for healthy subjects.

To address these limitations a new collection of both WL and NBI frames, indicated as the “Frame quality dataset” was added to the existing dataset. The recordings of the videolaryngoscopies (VLs) on 51 patients with a healthy larynx or affected by several laryngeal conditions (carcinoma, papillomatosis, polyps, Reinke’s edema, granulomas, and nodules) from the Units of Otolaryngology and Head and Neck Surgery of the IRCCS San Martino Hospital-University of Genova (Italy) and Hospital Clínic of Barcelona (Spain) were retrieved. The Institutional Review Board approval was obtained at both institutions (CER Liguria: 169/2022; Reg. HCB/2023/0897).

At both centers, the VLs were captured in the office using a flexible naso-pharyngo-laryngoscope (HD Video Rhino-laryngoscope Olympus ENF-VH, Olympus Medical System Corporation, Tokyo, Japan) through a transnasal route. As a general policy of the two centers, the VLs were performed first using WL and then switching to NBI.

VideoLAN VLC media player v.3.0.18 software was used for the automatic sampling of the frames from the VLs. A supervised strategy was used for a binary classification task so that the extracted frames were divided into two categories according to the following criteria: frames with a close and clear view of the larynx as a whole or of its subsites without artifacts alterations such as blur, saliva, reflexes or underexposure were classified as “informative”. All the other extracted frames were classified as “non-informative”. At the University of Genova, three junior otolaryngologists (AI; AT; AP) independently classified the images. Afterward, a senior laryngologist (GP) revised the classification and gave the final approval. Similarly at the University of Barcelona, three junior otolaryngologists (CS; LR; BA) labeled the frames while a senior laryngologist (IV) reviewed the dataset.

The resulting database, i.e. the internal dataset**,** obtained from the union of publicly available image collection [8] and the University of Genova dataset included a total of 5147 frames. It was reorganized with a patient-wise allocation method into 3126 frames for the training phase, 1317 frames for validation, and the remaining 704 frames (346 “informative” and 358 “Uninformative”) for testing. Data were then further augmented by applying 10% vertical and horizontal shifts, 10% zoom, rotations ranging from 0° to 10° and horizontal flip.

The dataset retrieved from the Hospital Clínic of Barcelona was used as an external set to validate the performance of the selected DL model on data acquired and annotated independently from the training set, allowing for testing the robustness of the proposed method. Data variability is associated with the geographical diversity of the involved population, the endoscopic technique, and the annotation process conducted by different physicians. The external testing set consisted of 646 NBI and WL laryngeal frames (informative, n = 306; uninformative, n = 340).

The breakdown and content of the dataset is reported in Table 1.

**Table 1:** Internal and external datasets collection

Datasets	Origin	No. of frames	Optical modality	Laryngeal conditions
Internal				
NBI-InfFrames [8]	Italy	720	NBI	Carcinoma
Frame quality	Italy	4427	WL + NBI	Carcinoma, benign lesions, healthy
External	Spain	646	WL + NBI	Carcinoma, benign lesions, healthy

*NBI* narrow band imaging, *WL* white light

### Deep learning models selection

The deployment of computer-aided systems in real-world settings demands the DL convolutional neural networks (CNNs) to be light in terms of architecture so that the number of mathematical operations required to process an input image, known as Floating Point Operations Per Second (FLOPS), remains low, allowing faster inference time and simpler hardware requirements. A shallow CNN comprising three convolutional blocks and an equivalent number of fully connected layers was built to this end. In each convolutional block, a 3 × 3 convolution was followed by a batch normalization operation and a Rectified Linear Unit (ReLU) activation. The final number of model parameters was less than 0.3 million.

On the other side, a basic architecture may not be able to extract sufficient features to correctly predict the informativeness of a frame. Therefore, to evaluate which neural networks could meet the conditions mentioned above, a careful analysis of the literature related to the processing of laryngeal endoscopic frames was conducted. This included the work by Yao et al. [7], who presented a very detailed comparison of the performance of various state-of-the-art networks. Based on their results, two other deeper state-of-the-art networks were explored in this work: ResNet-50 and MobileNetv2. The advantage of the former lies with the skip connections or “residual blocks” which allow for better gradient flow resulting in improved accuracy, while being a compact version of ResNet grants it to accelerate the inference process. On the other hand, MobileNetv2 is well-suited for mobile and embedded devices where computational means are limited, thanks to the use of depthwise separable convolutions. After modifying the top classification layer to output the probability of the frame belonging to each of the informative and uninformative classes, using a softmax function, ResNet-50 presented 23.8 million parameters, while MobileNetv2 had 2.4 million parameters.

### Self-supervised pretext task

Instead of the standard ImageNet-based pre-training, the pretext task strategy presented by Gidaris et al. [13] was adopted to speed up the training of the classification model and enhance its performance. The pretext strategy is a self-supervised learning process that divides the computer vision pipeline into two tasks: the real task, e.g., any classification or detection task for which there are not enough annotated data available, and the pretext task which enables self-supervised data visualization learning. For the pretext task strategy, the dataset was derived by randomly sampling 232 frames from the internal set. From these frames, patches measuring 512 × 512 pixels were cropped, extracted, and subjected to rotations of 0°, 90°, 180°, and 270°. The three networks were pre-trained for 200 epochs on the rotated patches to predict the correct rotation angle, with the categorical cross-entropy loss, the Adam optimizer, a batch size of 8, an initial learning rate of 0.0001 and the following learning rate decay rule:

$$lr=\frac{1}{1 + 0.9 \cdot \mathrm{ epoch}}\cdot {lr}_{0}.$$

The best-performing weights obtained during this phase were used to initialize the weights of the corresponding models for the real task, which involved training the CNN for 200 epochs using the same hyperparameters as those of the pretext task for the purpose of classifying the informative frames.

### Outcome evaluation

The outcomes of the DL models were evaluated by comparing the predicted classes with the ground-truth classes. True positives (TPs) represent the number of frames accurately predicted as belonging to the informative class, while true negatives (TNs) account for the number of uninformative frames correctly predicted. The false negatives (FNs) correspond to informative frames not identified by the model, and conversely, false positives (FPs) refer to frames wrongly categorized as part of that class. Based on these definitions, the models’ performance was assessed by calculating standard evaluation metrics such as precision, recall, F1-score, and Receiver Operating Characteristic (ROC) curve as previously reported and explained in the literature [4].

To provide better insights into the workings of the CNNs, the gradient-weighted Class Activation Mapping (gradCAM) algorithm was implemented [14]. This algorithm outputs a heatmap of the important regions in an image used by the network to make its predictions, by computing the gradients of the predicted class with respect to the final convolutional layer’s feature maps. It permits users to understand the DL model decisions, thereby facilitating debugging and improvements.

### Video testing

Four unedited preoperative videolaryngoscopies not used for the model’s training were selected for testing the computational speed of the model and simulating a real-time frame quality classification during an examination. In fact, in clinical practice, the assessment occurs through the analysis of videos, where temporal information also holds significant value. Thus, the informativeness of the selected video was analyzed in a dynamic context by measuring the Video Informativeness Level (VIL), which is equivalent to the fraction of frames predicted as informative with regard to the totality of examined frames:

$$VIL=\frac{number\,of\,video\,frames\,predicted\,as\,informative}{total\,number\,of\,frames}\%.$$

This measure can quantify the part of the video that is really relevant for the examiner with potential implications for selective data storage and a faster review process.

The classification of each frame is presented to the user as a colored border around the frame (green = informative, red = uninformative). To unlock the real-time processing of the proposed method, the ResNet-50_Pretext_ model was developed with the Keras library, compressed, and then saved in the ONNX format. This allowed the informativeness of any frame to be inferred in less than 0.020 s on average with an NVIDIA RTXTM A6000 GPU (48 GB of memory) and through different deep-learning frameworks (e.g., Tensorflow or Pytorch).

### Statistical analysis

Categorical variables are reported as frequencies and percentages, while continuous variables are reported as medians and first–third quartiles. The McNemar test was employed to statistically compare the performance of the three different CNNs on the internal test set. The diagnostic performance of the selected model was compared between the internal and external test sets by using the Receiver Operating Characteristic (ROC) curves and Area under the ROC Curve (AUC) calculations. Pairwise comparisons of AUCs were conducted using a Z-test [15, 16]. A two-sided p < 0.05 was considered significant for both tests. Statistical analysis was carried out using Python version 3.9 (packages scipy.stat and statmodels version 0.13.2).

## Results

### Internal dataset

**Fig. 1:**
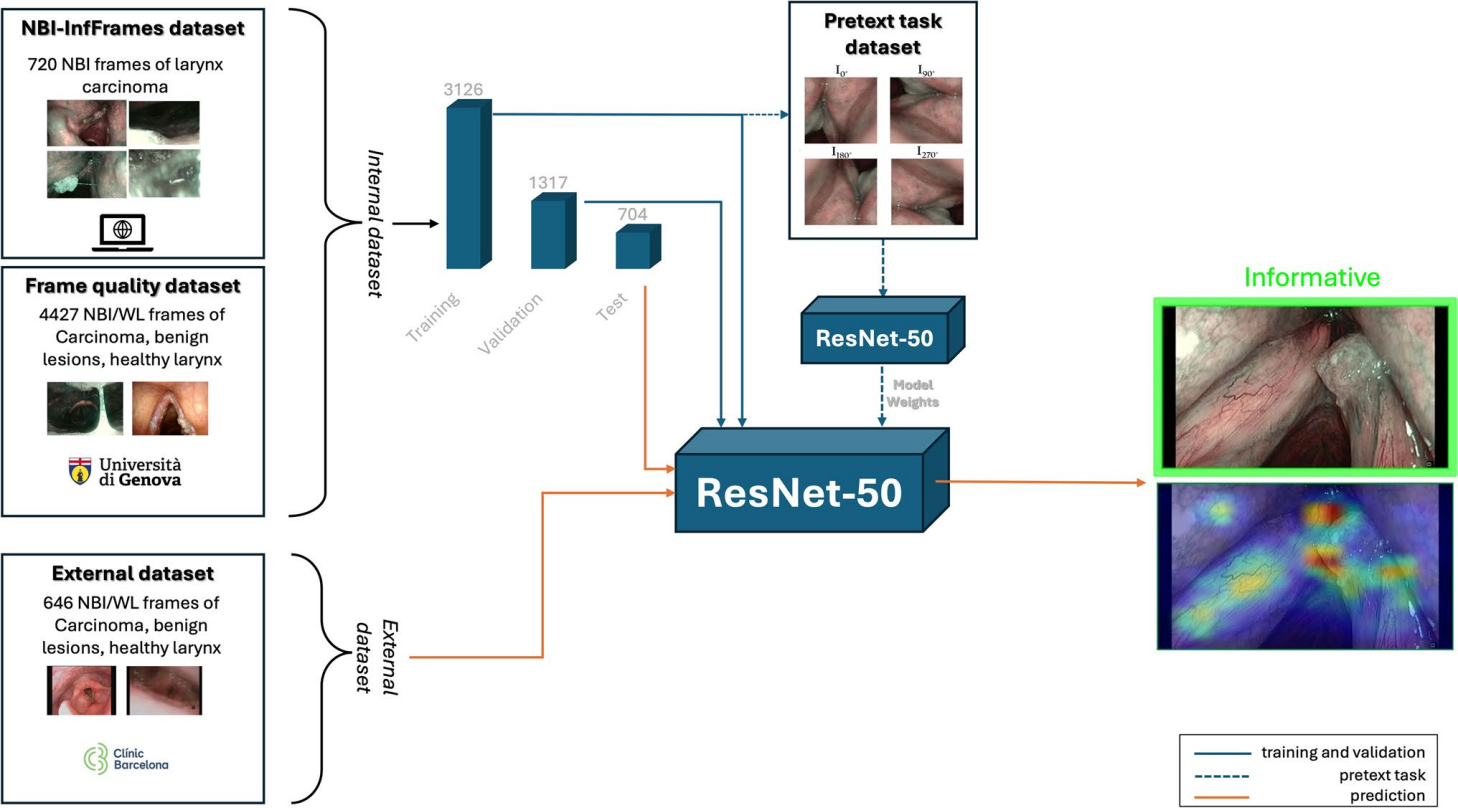
Flow chart of data utilization for the current dataset. *WL* white light, *NBI* narrow band imaging

**Fig. 2:**
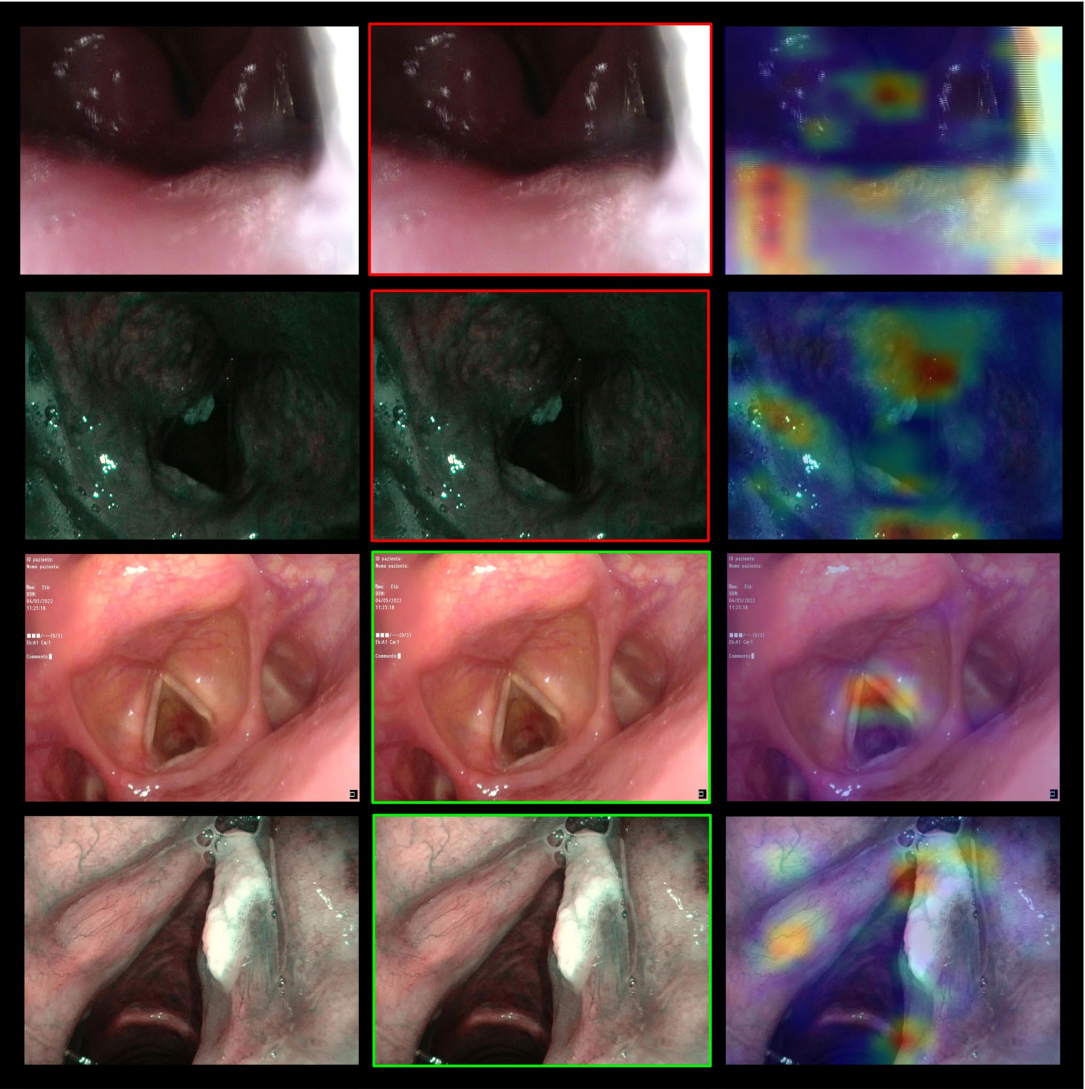
Examples of frames from the internal test set. In the first column, the original image is shown. In the second column, the prediction is output as a green frame for informative frames and as a red frame for uninformative frames. In the third column, the areas used by the model for the prediction are output according to a gradCAM heatmap

Figure 1 summarized the methodological process. The classification metrics generated by the three CNNs when tested on the internal test set (704 frames) after the pretext task-based weights initialization and model training are displayed in Table 2. The simple architecture of the shallow CNN often failed to recognize informative frames, revealing the lowest performance. Despite the ability to recognize more than 3/4 of the informative frames, the average F1 score was only 81%. Conversely, with MobileNetv2 the frames were classified with a precision and F1-score equal to 95%, while the recall was 96%. This comparison between the shallow CNN and MobileNetv2 confirms that a deeper architecture is necessary to extract the proper features for larynx inspection. However, the ResNet-50 model achieved the best performance during the internal testing phase of the classification model. Out of the 704 test frames, 673 were correctly classified, resulting in precision = 95%, recall = 97%, and the F1-score = 96%. Figure 2 shows some examples of the ResNet-50 model outputs.

**Table 2:** Performances of the deep learning models on the internal and external test sets

	True positives	True negatives	False positives	False negatives	Precision	Recall	F1score
Internal test set							
Shallow CNN	264 (76.3%)	313 (87.4%)	45 (12.6%)	82 (23.7%)	0.85	0.76	0.81
MobileNetv2	333 (96.2%)	339 (94.7%)	19 (5.3%)	13 (3.8%)	0.95	0.96	0.95
ResNet-50	**334 **(96.5%)	**339 **(94.7%)	**19 **(5.3%)	**12 **(3.5%)	**0.95**	**0.97**	**0.96**
External test set							
ResNet-50	272 (88.9%)	331 (97.4%)	9 (2.6%)	34 (11.1%)	0.97	0.89	0.93

The best performance values are shown in bold

The performances of the Shallow CNN vs. MobileNetv2 and Shallow CNN vs. ResNet-50 were significantly different (p-value < 0.05), while MobileNetv2 and ResNet-50 performed closely on the internal set. Given the superior F1 score, the ResNet-50 model was chosen for additional external test evaluation and video testing. Finally, an ablation study was conducted to validate the efficiency of the pretext task, demonstrating that the ResNet-50 F1-score improved by one percentage point when compared to the results achieved using the standard ImageNet-based weights.

### External dataset

**Fig. 3:**
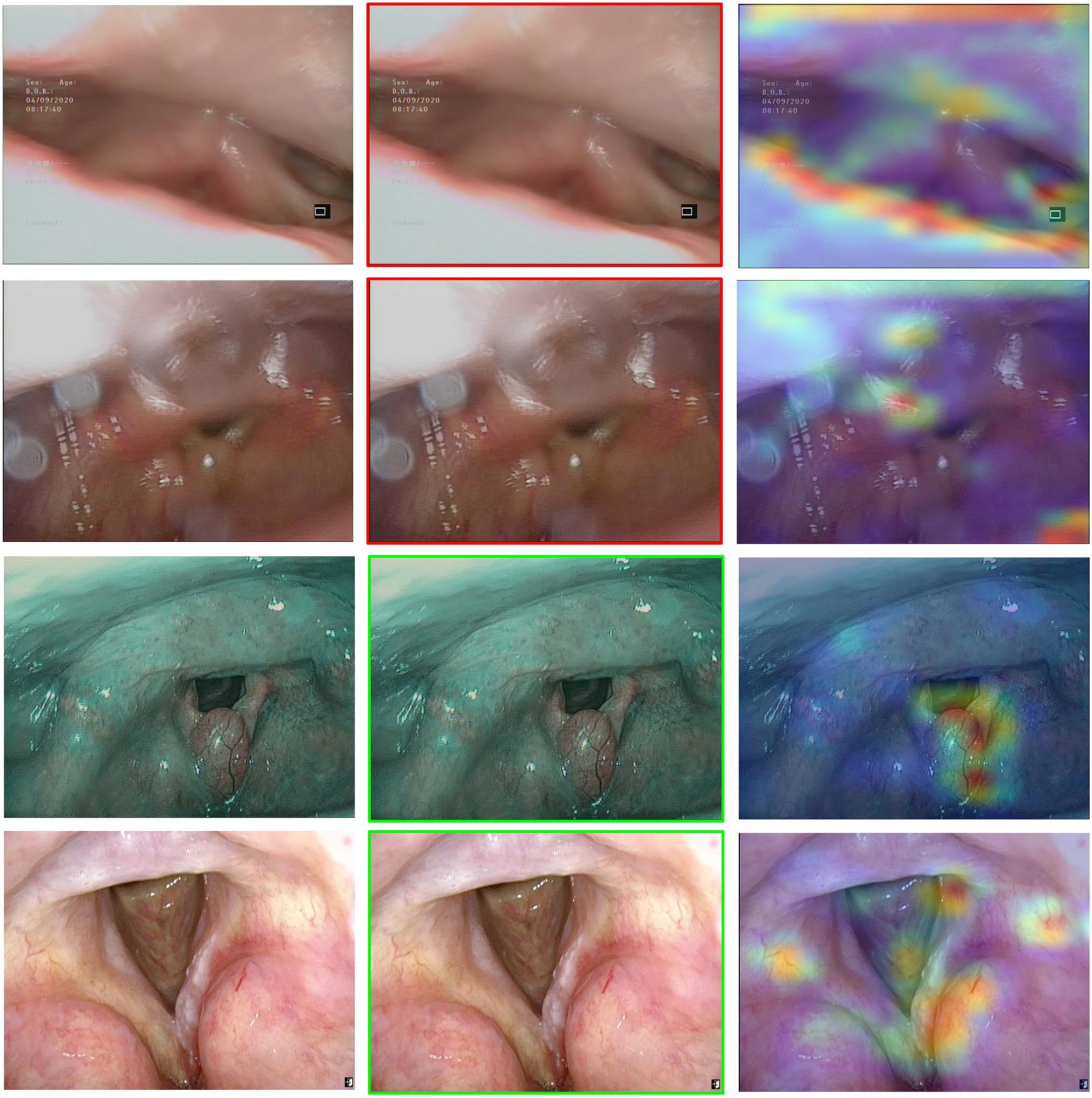
Examples of frames from the external test set. In the first column, the original image is shown. In the second column, the prediction is output as a green frame for informative frames and as a red frame for uninformative frames. In the third column, the areas used by the model for the prediction are output according to a gradCAM heatmap

**Fig. 4:**
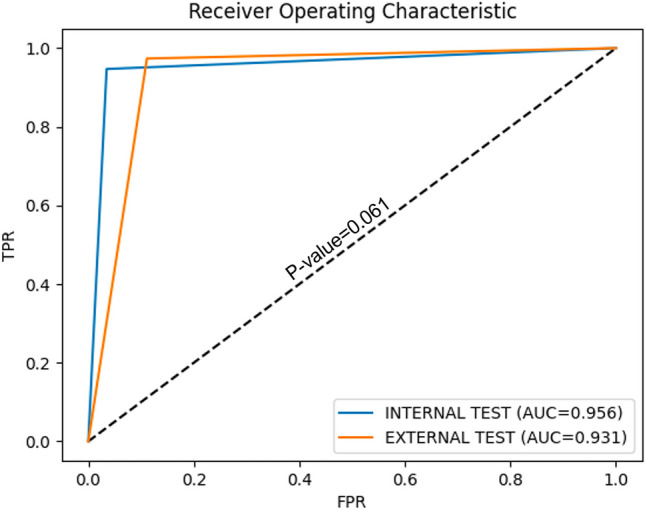
The model (ResNet-50, pre-trained with the pretext strategy) performance according to the receiver operating characteristics (ROC) curves for the internal (blue) and external (orange) test sets

Table 2 also reports the results of the pretext ResNet-50 model on the external set: the precision, recall, and F1-score were equal to 97%, 89%, and 93%, respectively. Figure 3 shows some graphical examples of the output of the pretext ResNet-50 model on the external test set. Figure 4 shows the pretext ResNet-50 model ROC curves for the internal and external sets. No statistical difference was found between the two datasets (p-value = 0.062).

### Video dataset

**Fig. 5:**
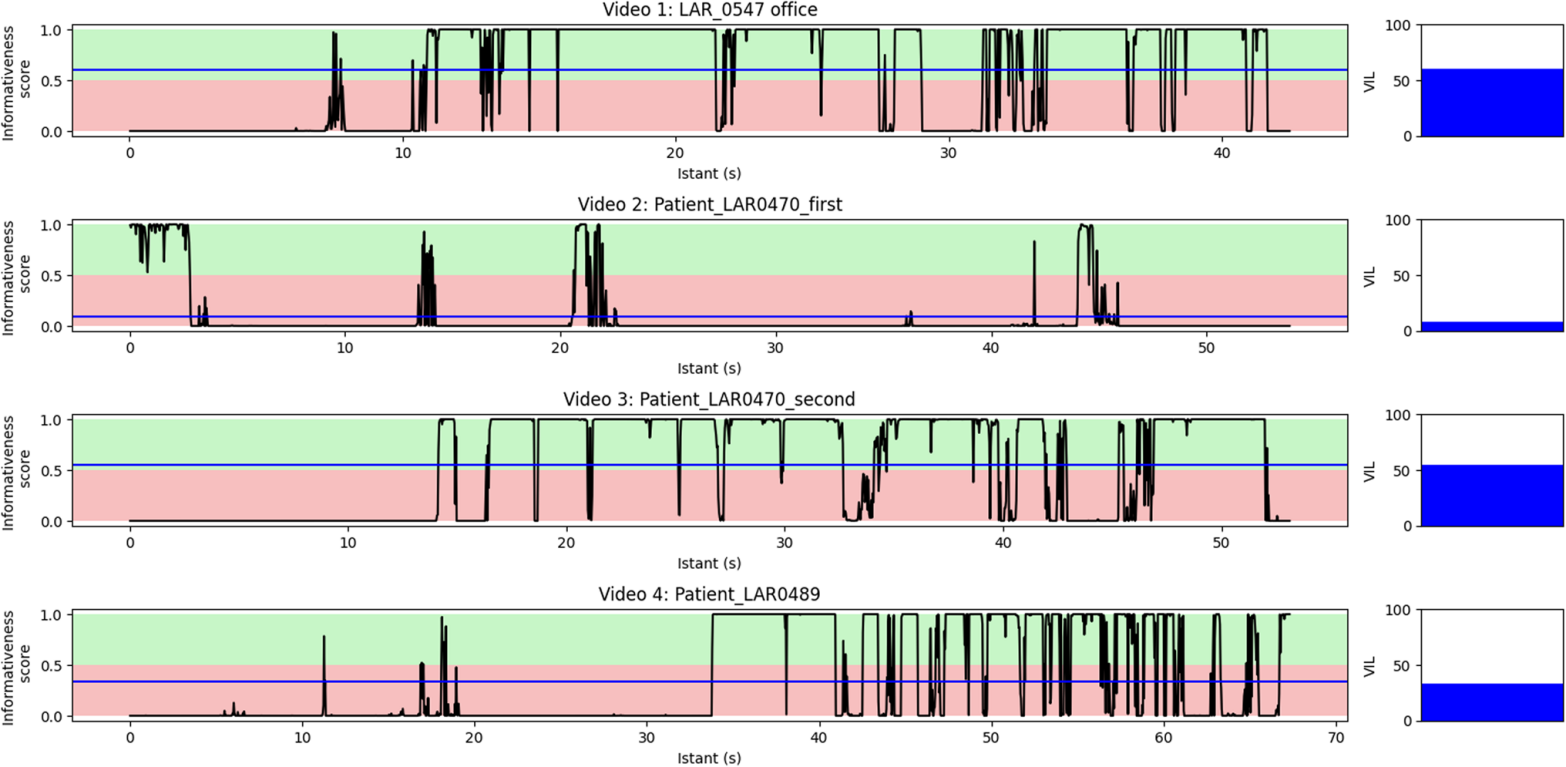
Plots of the model’s (ResNet-50, pre-trained with the pretext strategy) predictions trend during each test video. The black line represents the probability for the frame to be classified as “informative” (when in the green background) or “uninformative” (when in the red background). The blue line represents the average probability for the frames of the whole video to be assigned to the category “informative”. At the end of each bar plot a histogram with the Video Informativeness Level (VIL) value for the same video is reported. Videos 2 and 3 report the in-office diagnostic laryngoscopy of the same patient before and after local anesthesia

Table 3 resumes the video characteristics. With the current experiment hardware, the median computing times for the five videos was 0.015 [0.012–0.018] milliseconds per frame. Figure 5 shows the evolution of the Video Informativeness Level (VIL) during the different test videos. The four testing videos are provided in the supplemental materials.

**Table 3:** Characteristics of the tested laryngoscopy videos

ID	Length	Frames per second	Median inference time (s)	VIL (%)	Clinical setting
1	00′42′′	29.97	0.0155	60	In-office
2	00′53′′	29.97	0.0156	8	In-office
3	00′53′′	29.97	0.0155	55	In-office
4	01′07′′	29.97	0.0155	33	In-office

Inference times and the Video Informativeness Level (VIL) are calculated after applying the Res-Net50 deep learning model

## Discussion

The introduction of an automatic informative frame classifier offers several advantages, including the possibility of time savings during the review of endoscopic examinations, facilitating the clinical decision-making process, and the pre-selection of frames to label and process with computer-aided disease detection and diagnosis systems.

In this study, we tested the ability of several DL models (from a simple and lightweight shallow CNN to the state-of-the-art MobileNetv2 and ResNet-50 deeper networks) to classify informative frames. The latter proved itself capable of individuating good-quality frames with diagnostic relevance from laryngoscopic videos with real-time performance. This topic has been studied by a few publications in the literature so far. The publicly available small dataset of 720 frames by Moccia et al. [8] incorporated in our study was used by Patrini et al. [6] to test if an automated DL strategy could improve the accuracy of this task. Indeed, the CNN employed outperformed the original results achieved with a traditional machine learning approach, demonstrating that DL models should be the first choice in this field. Finally, on the same dataset, Galdran et al. [5] tried to use a similar DL strategy with a light-weight CNN achieving similar diagnostic performances but with less computational time paving the way for real-time implementation. Yao et al. [7] in 2022 investigated the same task on 22,132 laryngoscopic frames (healthy and with vocal fold polys patients) classifying them into informative or uninformative binary classification achieving comparable outcomes (Recall = 0.76–0.90; Precision = 0.63–0.94; F1-score = 0.69–0.92). The same group recently published a further step in this research, investigating the role of the previously developed algorithm in facilitating DL models development by automatically extracting the informative frames for training a DL classifier capable of diagnosing vocal fold polyps [17]. Their results showed that the classifier trained on machine-labeled frames held improved performance compared to the classifier trained on human-labeled frames. The latter experience shows that the algorithm present in this paper can also have a role in the development of future DL models.

Nevertheless, in all these studies, no attempt was made to implement these algorithms on videoendoscopies. In the present work, we tested our DL model on videos simulating clinical use by developing a user interface capable of classifying in real time the informativeness of the video frames. This enables providing immediate feedback to the clinician during the endoscopy. At the end of each video, the Video Informativeness Level (VIL) never exceeded 60% suggesting that our system could potentially reduce the size of the data to be stored and subsequently reviewed.

**Fig. 6:**
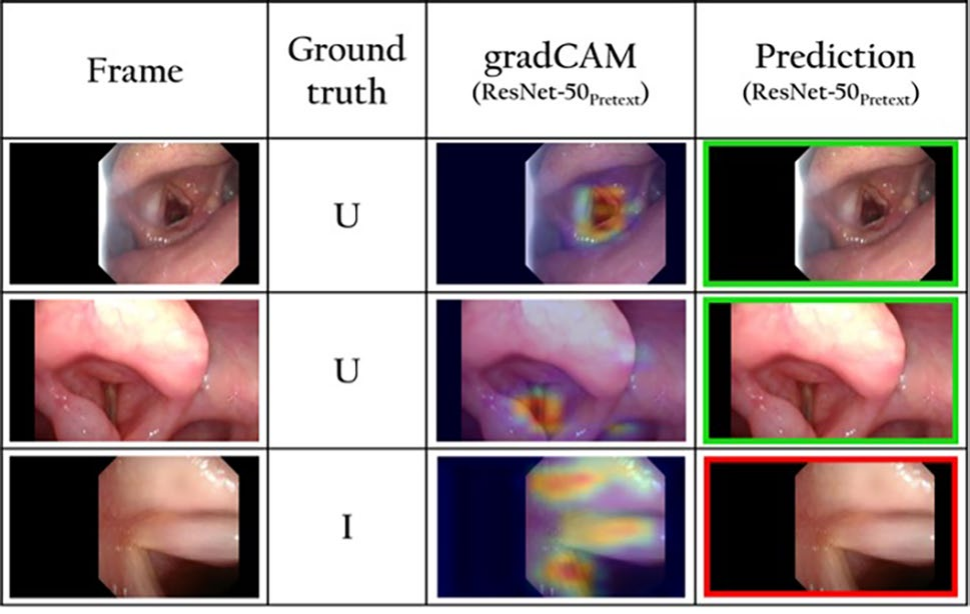
Examples of miss-predicted frames. The first column shows the original frames. The second column shows how the frame was originally labeled by the physician as “I” (informative) or “U” (uninformative). In the third column, the areas considered by the model for the prediction are output according to a gradCAM heatmap. In the fourth column, the prediction is output as a green frame for informative frames and as a red frame for uninformative frames

The video tests showed that, even though the model did not achieve perfect recall and precision outcomes, the algorithm implementation is clinically reliable. Moreover, misclassification cases are largely imputable to the criteria used during the frames labeling process due to the highly subjective evaluation as there is no gold standard to assess the quality of a frame. As demonstrated in Fig. 6, frames in which there is a slight blur but not enough to set the ground truth as uninformative (e.g. third row), as well as those that exhibit reflections along with a clear view of the vocal cords (e.g. first and second rows), can be borderline cases. Furthermore, the real-time frame classification can also be implemented to help improve the quality of endoscopic examinations, informing clinicians about the quality of the collected video and allowing them, for instance, to identify the moments during the endoscopy that should be submitted to a classification algorithm to predict lesions’ histology [12, 18, 19].

## Conclusions

In this study, we introduce an automated approach for assessing frame quality during laryngoscopy examinations. Notably, our work establishes the first extensively generalized multi-center and multi-modality dataset designed for this specific purpose. The proposed DL method demonstrated superior performance in identifying diagnostically relevant frames within laryngoscopic videos. With its solid accuracy and real-time capabilities, the system stands poised for deployment in clinical settings, either autonomously for objective quality control or in conjunction with other algorithms within a comprehensive AI toolset aimed at enhancing tumor detection and diagnosis. The next phase of our research will involve subjecting the system to clinical trials, aiming to validate its effectiveness and reliability in real-world medical scenarios.

### Supplementary Information

Below is the link to the electronic supplementary material.

Supplementary file1 Video S1: In this endoscopic examination of the upper aerodigestive tract, the oropharynx is initially shown, and finally the larynx and trachea are visible. On the right vocal fold, a leukoplakia with abnormal vascularity on Narrow Band Imaging (NBI) is visible. The informativeness of every frame is analyzed by the model and the visual result is shown with a green or red frame. The Video Informativeness Level (VIL) is reported in the upper right corner. (MP4 64298 KB)

Supplementary file2 Video S2: This endoscopy shows a white light examination of a right laryngeal squamous cell carcinoma. The patient shows a low compliance to the examination and the glottis is not visible. The informativeness of every frame is analyzed by the model and the visual result is shown with a green or red frame. The Video Informativeness Level (VIL) is reported in the upper right corner. (MP4 67107 KB)

Supplementary file3 Video S3: In this video, the same patient of video 3 was re-examined after having administrated topical local anesthesia with 2–3 ml of 5% neoblucaine administered transnasally. Therefore, the examination of the laryngeal box is much more accurate. The informativeness of every frame is analyzed by the model and the visual result is shown with a green or red frame. The Video Informativeness Level (VIL) is reported in the upper right corner. (MP4 66974 KB)

Supplementary file4 Video S4: This videoendoscopy shows a recurrent squamous cell carcinoma of the right vocal fold, both in white light and Narrow Band Imaging (NBI). The recording starts from the nasopharynx and shows the oropharynx, the larynx, and finally the upper part of the trachea. The informativeness of every frame is analyzed by the model and the visual result is shown with a green or red frame. The Video Informativeness Level (VIL) is reported in the upper right corner. (MP4 86493 KB)

## Data Availability

The data that support the findings of this study are available from the corresponding author upon reasonable request.

## References

[1] Piazza C, Cocco D, de Benedetto L et al (2010) Narrow band imaging and high definition television in the assessment of laryngeal cancer: a prospective study on 279 patients. Eur Arch Oto-Rhino-Laryngol 267(3):409–414. 10.1007/S00405-009-1121-6 10.1007/S00405-009-1121-6 19826829

[2] Vilaseca I, Valls-Mateus M, Nogués A et al (2017) Usefulness of office examination with narrow band imaging for the diagnosis of head and neck squamous cell carcinoma and follow-up of premalignant lesions. Head Neck 39:1854–1863. 10.1002/HED.24849 28640478 10.1002/HED.24849

[3] Haug CJ, Drazen JM (2023) Artificial intelligence and machine learning in clinical medicine, 2023. N Engl J Med 388:1201–1208. 10.1056/NEJMRA2302038 36988595 10.1056/NEJMRA2302038

[4] Sampieri C, Baldini C, Azam MA et al (2023) Artificial intelligence for upper aerodigestive tract endoscopy and laryngoscopy: a guide for physicians and state-of-the-art review. Otolaryngol Head Neck Surg 169:811–829. 10.1002/OHN.343 37051892 10.1002/OHN.343

[5] Galdran A, Costa P, Campilho A (2019) Real-time informative laryngoscopic frame classification with pre-trained convolutional neural networks. Proc Int Symp Biomed Imag 2019:87–90. 10.1109/ISBI.2019.8759511 10.1109/ISBI.2019.8759511

[6] Patrini I, Ruperti M, Moccia S et al (2020) Transfer learning for informative-frame selection in laryngoscopic videos through learned features. Med Biol Eng Comput 58:1225–1238. 10.1007/s11517-020-02127-7 32212052 10.1007/s11517-020-02127-7

[7] Yao P, Witte D, Gimonet H et al (2022) Automatic classification of informative laryngoscopic images using deep learning. Laryngosc Investig Otolaryngol 7:460–466. 10.1002/lio2.754 10.1002/lio2.754 PMC9008155 35434326

[8] Moccia S, Vanone GO, De ME et al (2018) Learning-based classification of informative laryngoscopic frames. Comput Methods Progr Biomed 158:21–30. 10.1016/j.cmpb.2018.01.030 10.1016/j.cmpb.2018.01.030 29544787

[9] Azam MA, Sampieri C, Ioppi A et al (2022) Deep learning applied to white light and narrow band imaging videolaryngoscopy: toward real-time laryngeal cancer detection. Laryngoscope 132:1798–1806. 10.1002/lary.29960 34821396 10.1002/lary.29960 PMC9544863

[10] Sampieri C, Azam MA, Ioppi A et al (2024) Real-time laryngeal cancer boundaries delineation on white light and narrow-band imaging laryngoscopy with deep learning. Laryngoscope. 10.1002/LARY.31255 38174772 10.1002/LARY.31255

[11] Azam MA, Sampieri C, Ioppi A et al (2022) Videomics of the upper aero-digestive tract cancer: deep learning applied to white light and narrow band imaging for automatic segmentation of endoscopic images. Front Oncol 12:900451. 10.3389/fonc.2022.900451 35719939 10.3389/fonc.2022.900451 PMC9198427

[12] Dunham ME, Kong KA, McWhorter AJ, Adkins LK (2022) Optical biopsy: automated classification of airway endoscopic findings using a convolutional neural network. Laryngoscope 132:S1–S8. 10.1002/lary.28708 32343434 10.1002/lary.28708

[13] Gidaris S, Singh P, Komodakis N (2018) Unsupervised representation learning by predicting image rotations. international conference on learning representations

[14] Selvaraju RR, Cogswell M, Das A et al (2020) Grad-CAM: visual explanations from deep networks via gradient-based localization. Int J Comput Vis 128:336–359. 10.1007/s11263-019-01228-7 10.1007/s11263-019-01228-7

[15] Hanley JA, McNeil BJ (1982) The meaning and use of the area under a receiver operating characteristic (ROC) curve. Radiology 143:29–36 7063747 10.1148/radiology.143.1.7063747

[16] Hanley JA, McNeil BJ (1983) A method of comparing the areas under receiver operating characteristic curves derived from the same cases. Radiology 148:839–843. 10.1148/radiology.148.3.6878708 6878708 10.1148/radiology.148.3.6878708

[17] Yao P, Witte D, German A et al (2023) A deep learning pipeline for automated classification of vocal fold polyps in flexible laryngoscopy. Eur Arch Otorhinolaryngol 1:1–8. 10.1007/S00405-023-08190-8/FIGURES/5 10.1007/S00405-023-08190-8/FIGURES/5 37695363

[18] Cho WK, Lee YJ, Joo HA et al (2021) Diagnostic accuracies of laryngeal diseases using a convolutional neural network-based image classification system. Laryngoscope 131:2558–2566. 10.1002/lary.29595 34000069 10.1002/lary.29595

[19] You Z, Han B, Shi Z et al (2023) Vocal cord leukoplakia classification using deep learning models in white light and narrow band imaging endoscopy images. Head Neck 45:3129–3145. 10.1002/HED.27543 37837264 10.1002/HED.27543

